# EMS1/DLL4-Notch Signaling Axis Augments Cell Cycle-Mediated Tumorigenesis and Progress in Human Adrenocortical Carcinoma

**DOI:** 10.3389/fonc.2021.771579

**Published:** 2021-11-10

**Authors:** Yu-Gang Huang, Ya Wang, Rui-Juan Zhu, Kai Tang, Xian-Bin Tang, Xiao-Min Su

**Affiliations:** ^1^ Department of Pathology, Taihe Hospital, Hubei University of Medicine, Shiyan, China; ^2^ Department of Immunology, Nankai University School of Medicine, Tianjin, China; ^3^ Department of Pediatric, Taihe Hospital, Hubei University of Medicine, Shiyan, China

**Keywords:** adrenocortical carcinoma, ACC, ESM1, DLL4, Notch signaling pathway

## Abstract

Adrenocortical carcinoma (ACC) is a rare malignant neoplasm that is prone to local invasion and metastasis. Meanwhile, overexpressed endothelial cell-specific molecule 1 (*ESM1*) is closely related to tumorigenesis of multitudinous tumors. However, the prognosis value and biological function of *ESM1* in ACC remains undefined. In the current essay, the assessment in human ACC samples and multiple public cancer databases suggested that ESM1 was significantly overexpressed in ACC patients. The abnormal expression of *ESM1* was evidently correlated with dismal overall survival (OS) in ACC patients. Then, the gene-set enrichment analysis (GSEA) was applied to unravel that *ESM1* was mostly involved in cell cycle and Notch4 signaling pathway. Furthermore, *in vitro* experiment, RNA interference of *ESM1* was carried out to state that ESM1 augments CDK1 and p21-mediated G2/M-phase transition of mitosis, cell proliferation *via* DLL4-Notch signaling pathway in human ACC cell line, SW13 cells. Additionally, two possible or available therapeutic strategies, including immunotherapy and chemotherapy, have been further explored. Immune infiltration analysis highlighted that no significant difference was found in ACC patients between EMS1^high^ and EMS1^low^ group for immune checkpoint-related genes. In addition, the overexpression of *ESM1* might trigger the accumulation of tumor mutation burden (TMB) during the cell cycle of DNA replication in ACC. The gene-drug interaction network then indicated that ESM1 inhibitors, such as cisplatin, might serve as potential drugs for the therapy of ACC. Collectively, the results asserted that *ESM1* and related regulators might act as underlying prognostic biomarkers or novel therapeutic targets for ACC.

## 1 Introduction

Adrenocortical carcinoma (ACC) is an aggressive and rare malignant tumor derived from the adrenal cortex and accounting for ~14% of primary adrenal tumor (1). The ACC mainly occurs in childhood and adults (40–60 years old) with a bimodal age distribution (2, 3). The 5-year survival rate is about 35% for ACC with locally advanced stages and less than 28% for ACC with metastases (4, 5). It indicates that ACC reveals high tumor heterogeneity and harbors a dismal prognosis. Thus far, effective therapeutic strategies for the treatment of ACC are scarce. From 1914 to now, surgery and mitotane combined with platinum-based chemotherapy are still the main available therapeutic strategies (1). Multiple biomarkers related to the diagnosis, prognosis, or therapy of ACC have been reported. Molecular literatures have shown that the most common driver genes for ACC samples were *TP53* (6, 7) and *CTNNB1* (8) mutations. Sbiera et al. demonstrated that *SOAT1* may act as a molecular target of mitotane in ACC (9). Accumulative studies have proposed that inhibition of WNT signaling pathway (10), downregulation of p53-RB (11), and abnormal maintenance of telomere [*ATRX* (12, 13), *DAX* (14), and *TERT* (8, 15)] were closely associated with dismal prognosis of ACC. However, all the research-related above biomarkers of ACC are still in the stage of preclinical research. Thus, it is urgent and necessary to uncover potential biomarkers to evaluate the prognostic value in early stage or impolder novel therapeutic strategies.

Endothelial cell-specific molecule 1 (ESM1), also named as endocan, is a secreted protein mainly expressed in the endothelial cells, such as human lung and kidney tissues (16). The expression of ESM1 can promote angiogenesis, affect vascular permeability, promote cell division, and affect the regulation of cell cycle (17). The expression of ESM1 is mostly detected in endothelium cells (18) and is prominently upregulated in multiple neoplasms including hepatocellular carcinoma (19), bladder cancer (20), head and neck squamous cell carcinoma (HNSCC) (21), colorectal cancer (22), nonsmall cell lung cancer (NSCLC) (23), etc. Additionally, serum endocan also could be detected in a crowd of patients with cancers (24–26). However, whether ESM1 is aberrantly expressed and related biological processes have not been investigated and elucidated in ACC patients.

In this essay, the analysis of the database, Gene Expression Omnibus (GEO) GEO and Cancer Genome Atlas (TCGA) databases, coupled with the immunohistochemistry (IHC) detection of human specimens and cell line experiment, suggested that ESM1 was significantly overexpressed, associated with poor prognosis, involved in Notch signaling pathway-mediated cell cycle in ACC. This study may help us to more comprehensively understand the expression pattern and prognostic value of ESM1 in ACC patients and gain further insight into the diagnosis, occurrence, or development of ACC.

## 2 Materials and Methods

### 2.1 Human ACC Specimens

Formalin-fixed and paraffin-embedded (PPFE) specimens were collected from patients with ACC and adrenocortical adenoma and stored at room temperature (20°C~25°C), from August 2011 to August 2021 at the Department of Pathology, Taihe Hospital. According to the pathological features, at least two pathologists diagnosed all PPFE specimens and reached an agreement. Lastly, this study included six cases of ACC, 12 cases of adrenocortical adenoma, and 12 cases of normal adrenal cortex specimens (adjacent to adrenocortical adenoma). Details of these involved patients are shown in **Supplementary Table S1**.

### 2.2 IHC Staining and Scoring

According to the manufacturer’s protocol, hematoxylin-eosin (HE) and immunohistochemistry (IHC) staining of PPFE samples were performed. Firstly, 3 μm of ACC specimens, adrenocortical adenoma specimens, and normal adrenal cortex specimens were sliced from the PPFE. For HE, tissue sections were stained with hematoxylin-eosin staining kit (E607318-0200, Sangon, Shanghai, China) for morphological observations. For IHC, all slices were dewaxed with xylene and rehydrated with graded ethanol. Endogenous peroxidase activity was blocked by 3% hydrogen peroxide in methanol for 10 min. The antigen was recovered in a pressure cooker with EDTA at pH 9.0 for 4 min. After washing in PBS three times (3 min each time), the sections were incubated with primary antibody (**Supplementary Table S2**) for 1 h at 37°C. After incubation with HRP-labeled second antibody at 37°C for 0.5 h, hematoxylin staining was performed at 37°C for 30 s.

The ESM1-positive cells were counted in five random high-power fields (×400), and the average positive cell ratio was calculated. Then, a combined score of ESM1 was conducted to evaluate the intensity and distribution of ESM1 cytoplasmic and membrane staining. The ESM1-positive cells of cytoplasmic staining was assigned a score of 0 (no discernible ESM1 staining), 1 (1%~49%), 2 (50%~79%), or 3 (≥80%). The ESM1-positive cells of membrane staining were graded as follows: a score of 0 (absent), 1 (1%~19%), 2 (20%~49%), or 3 (≥50%). The two scores were added up to yield an overall score of 0 to 6. Thus, the combined score denoted the expression level of ESM1. The IHC staining was independently scored by three authors in a blinded manner. For the score difference among observers, the consistency can be evaluated using a multihead microscope, and then the final score can be recorded.

### 2.3 Bioinformatics Verification of *ESM1* Expression

As further validated, the two open access disease genomics databases, including the Gene Expression Omnibus (GEO) of NCBI and Gene Expression Profiling Interactive Analysis (GEPIA) were utilized to analyze ESM1 expression in ACC patients. In this essay, two GEO datasets, namely GSE90713 and GSE19750, were retrieved for the analysis of adrenocortical carcinoma (**Supplementary Table S3**). As an interactive online service platform, GEPIA is then widely used to analyze gene expression of tumors and normal samples from the TCGA and Genotype-tissue Expression dataset (GTEx) projects (27). Hence, the GEPIA dataset was performed to detect the mRNA expression of *ESM1* between ACC (*n* = 79) and normal tissues (*n* = 128), and in different pathological stages.

### 2.4 Correlation Between *ESM1* Expression and Clinicopathological Signatures in ACC

The correlation between *ESM1* and clinicopathological features was then studied based on TCGA-ACC dataset. The 79 ACC samples in the expression matrix were divided to two groups, including 39 ACC samples with *ESM1* low expression (ESM1^low^) and 40 ACC samples with *ESM1* high expression (ESM1^high^) by the median cutoffs. The risk types were grouped by ‘ggrisk’ package of R software (version 4.0.3) (28, 29). Sanguini diagram was drawn *via* the ‘ggalluval’ package (30) for displaying the distribution of the gene expression in survival status, ages, genders, stages, and other clinical characteristics for ACC.

The prognosis analysis, including overall survival (OS) and progression-free survival (PFS) were performed in ACC with ESM1^high^ and ESM1^low^ by ‘survival’ and ‘survminer’ packages (28). The ROC curve was conducted *via* ‘ pROC’ and ‘ ggplot2’ packages (31) to predict the specificity and sensitivity of *ESM1* expression in ACC patients. The univariate (uni-Cox) and multivariate Cox (multi-Cox) regression analysis was analyzed and applied to develop the nomogram. The *p-values*, HR, and 95% confidence interval (CI) of each variable *via* ‘forestplot’ R package were shown by forest. Based on the results of the multi-Cox analysis, The nomogram (32) was constructed to provide a graphical representation of the risk factors and calculate the 1-, 2-, and 3-year overall recurrence for an ACC patient *via* ‘rms’ R package (33).

### 2.5 Gene-Set Enrichment Analysis in ACC

The gene-set enrichment analysis (GSEA) software 4.0.3 (Broad Institute, Cambridge, MA, USA) was applied to probe and uncovered biological mechanisms of ESM1 in ACC patients based on TCGA datasets (34, 35). The three predefined gene sets from the Molecular Signatures Database were analyzed, including ‘c2.cp.kegg.v7.2.symbols.gmt’, ‘h.all.v7.2.symbols.gmt’, and ‘c2.cp.biocarta.v7.2.symbols.gmt’. Normalized enrichment scores (NES) were reckoned as the main GSEA statistic results. Statistical significance threshold was set as |NES|>1, normalized *p*-values (NOM *p*-values) <0.05, and FDR <0.25.

### 2.6 Analysis of Immune Cell Infiltration Profile and Correlation

As one of the crucial indicators to predict the effect of immunotherapy, the immune cell infiltration in tumor has become a research hotspot (36). The two online service platforms, CIBERSORT (37) and TIMER (31), were carried out to analyze immune cell infiltration based on TCGA-ACC dataset. The expression matrix from TCGA dataset was normalized through ‘Limma’ package of R software. The gene expression of immune checkpoint, including *CD274*(*PD-L1)*, *CTLA4*, *HAVCR2*, *LAG3*, *PDCD-1*(*PD-1)*, *PDCD1LG2*(*PD-L2)*, *TIGIT*, and *SIGLEC15(CD33L3)* were closely related to immunotherapy, were investigated in ACC patients with ESM1^high^ and ESM1^low^ (38). Moreover, the Tumor Immune Dysfunction and Exclusion (TIDE) algorithm was performed *via* ‘ggplot2’ and ‘ggpubr’’ packages to predict the potential immunotherapeutic response (39). The higher the TIDE score was, the worse efficacy of immune checkpoint blocking therapy (ICB) was. In order to predict antitumor effect of PD-1/PD-L1 mono-antibody, the ‘ggstatsplot’ package of R software were further conducted to analyze to the correlation between *ESM1* expression and the microsatellite instability (MSI) or tumor mutation burden (TMB) (40). The correlation between quantitative variables without a normal distribution was described by Spearman’s correlation analysis.

### 2.7 Gene-Drug Interaction Network Analysis

The gene-drug interaction network of *ESM1* was constructed *via* the Comparative Toxicogenomics Database (CTD) (41) for chemotherapeutic drugs that could reduce or elevate the mRNA or protein expression levels of *ESM1*. Briefly, the *ESM1* were searched in the CTD database, and the gene-drug interaction network was visualized by the OmicShare tools.

### 2.8 Human Cell Line Experiment

#### 2.8.1 siRNA Experiment

The siRNA treatment was conducted as previously described (42). The human SW13 cell, one of the human ACC cell lines, was transfected with 50 nmol of ESM1 siRNA (siESM1) or negative control siRNA (siNC) in a special medium (CM0451, Procell, Wuhan, China) for 48 h. SW13 cells were then lysed by TRIzol reagent (Invitrogen, Waltham, MA, USA) for total RNA isolation. The cDNA was obtained by oligo-dT primers and reverse transcriptase kit (Invitrogen, USA). Quantitative real-time PCR (qRT-PCR) was performed by SYBR Green PCR Master Mix (Qiagen, Hilden, Germany) and specific primers in an ABI Prism 7500 analyzer (Applied Biosystems, Waltham, MA, USA). GAPDH was an endogenous reference gene. Three replicates were set for all reactions. The 2^−ΔΔCt^ method was applied to calculate the relative expression of *ESM1* in ACC samples. The related primers are listed in **Supplementary Table S4**.

#### 2.8.2 Western Blotting

The human SW13 cells were transfected with ESM1 siRNA or control siRNA for 72 h. The whole protein from cell lysates was prepared for SDS-PAGE electrophoresis and was then transferred to PVDF membranes (Millipore, Burlington, MA, USA). The PVDF membranes were incubated with primary antibody (**Supplementary Table S2**) overnight at 4°C, washed by a washing buffer four times and then incubated with the secondary HRP-labeled antibody for 1 h at room temperature. After being washed for four times by a washing buffer, the membranes were detected using an enhanced chemiluminescence assay with Lumi-Glo reagents (Millipore, USA).

#### 2.8.3 Cell Proliferation Assay

Human SW13 cells were seeded in 96-well plates at 100 μl (total 1 × 10^4^ cells) and transfected with 50 nmol of siESM1 or siNC, then added 10 μl CCK-8 solution (Beyotime, Haimen, China) to each well. The cells were incubated for 72 h. The absorbance [optical density (OD)], representing the density of cells, was measured at 450 nm.

#### 2.8.4 Cell Migration Assay

Wound healing experiment were performed to analyze cell migration. Human SW13 cells were plated in 12-well plates in Leibovitz’s L-15 (PM151013, Procell, China) with 10% fetal bovine serum (FBS) and transfected with 50 nmol of siESM1 or siNC for 48 h. Twenty-microliter pipette tips were utilized to make wounds. Then each well was washed five times by PBS to remove the floating cells, and 3 ml Leibovitz’s L-15 (10% FBS, 1% antibiotic-antimycotic) was subjected. The scratch areas were photographed at 0, 24, 48, and 72 h.

#### 2.8.5 Cell Adhesion Assay

Human SW13 cells were seeded in 12-well plates (1 × 10^5^ cells/ml) and transfected with 50 nmol of siRNAs for 48 h. Cells were then transferred to 24-well plates for 3 h. Cells were rinsed and fixed with 4% PFA for 20 min. Then, cells were stained by Crystal Violet Staining kits (Beyotime, China) and incubated for 10 min. Cells adhered to the stroma were photographed and counted by Image J software.

#### 2.8.6 Transwell Assay

Transwell assay was performed to assess tumor cell invasion. Human SW13 cells were transfected with siESM1 or control siRNA for 48 h and plated in TranswellR cell culture chambers (Corning, Corning, NY, USA) with 1 × 10^4^ cells/well. The upper chamber of a TranswellR insert was filled with cell suspension. High concentration FBS (20% FBS), as a chemoattractant, was subjected to the lower chamber for 24 h. The cells under the membrane were fixed with 4% PFA and stained with crystal violet. Cells were photographed and counted by Image J software in five random fields per chamber.

#### 2.8.7 Cell Apoptosis Analyses

Human SW13 cells were transfected with siESM1 or control siRNA for 48 h. Cells were then disposed with Annexin V-FITC kit (Beyotime Biotechnology, China) and analyzed with flow cytometry (FACSCalibur, Bio-Rad, Hercules, CA, USA) to detect cell apoptosis. Data were analyzed using FlowJo7.6 software.

#### 2.8.8 Cell Cycle Assay

Cell cycle assay kit (ab112116, Abcam, Cambridge, MA, USA) was utilized for cell cycle assay. After transfection with siESM1 or siNC for 48 h, human SW13 cells were harvested and fixed in 70% ice-cold ethanol overnight at 4°C. Cells were then stained with RNase A (10 mg/ml) and propidium iodide (50 mg/mL) and analyzed by flow cytometer. Lastly, data were analyzed by FlowJo7.6 software.

### 2.9 Statistical Analysis

Statistical analysis was finished by SPSS 22.0 (IBM SPSS Inc., Chicago, IL, USA) and visualized by GraphPad Prism 9.0 (San Diego, CA, USA) software. A comparison of two groups of data was performed for the analysis of Student’s *t*-test. *p* < 0.05 was set as the statistical threshold. The meaning of symbols in this study were as follows: ns denotes no significant difference, *p < 0.05, ** *p* < 0.01, *** *p* < 0.001, **** *p* < 0.0001.

## 3 Results

### 3.1 ESM1 Is Overexpressed in ACC, Compared With Adrenocortical Adenoma or Normal Adrenal Cortex Tissues

**Figure 1:**
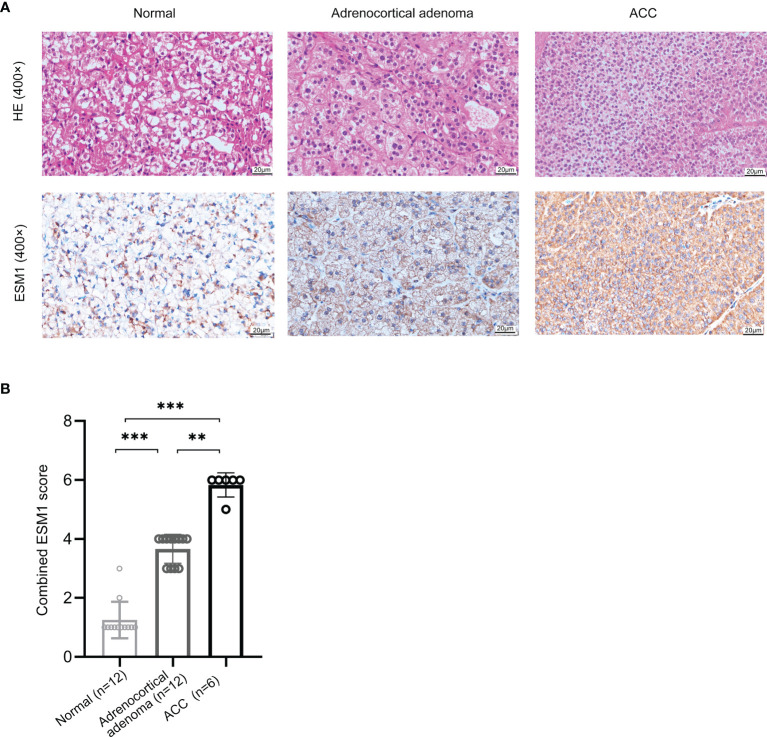
ESM1 is overexpressed in ACC patients, compared with normal adrenocortical samples or adrenocortical adenoma. **(A)** IHC for HE (upper) and ESM1 protein expression (bottom) in normal adrenal cortex, adrenocortical adenoma, and ACC samples (high power fields, ×400). **(B)** Combined ESM1 score in ACC, adrenocortical adenoma, or normal adrenal cortex tissues. **p < 0.01, ***p < 0.001.

In normal adrenal cortex, adrenocortical adenoma (benign tumor), and ACC (malignant tumor), IHC staining suggested that ESM1 was diffuse cytoplasmic and membrane positive in ACC. In adrenocortical adenomas, ESM1 was mainly cytoplasmic positive and faint membrane positive. In normal adrenal cortical tissues, only ESM1 cytoplasmic positive was found, and the proportion was relatively low, and ESM1 membrane positive was rare or absent (**Figure 1A**). In ACC, the combined ESM1 score was significantly higher than that in adrenocortical adenoma and normal adrenal tissues (*p* < 0.001, **Figures 1A, B**). It suggested that the ESM1 was strongly expressed in the cytoplasm and membrane of ACC. In adrenocortical adenomas, ESM1 was a strong cytoplasmic expression and weak membrane expression. It was weakly or moderately expressed in the cytoplasm of normal adrenal cortex cells.

**Figure 2:**
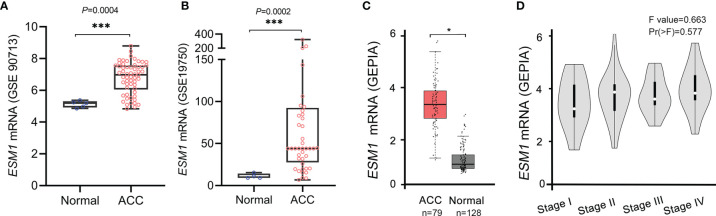
Based on GEO and TCGA datasets, *ESM1* was overexpressed in ACC patients, compared with normal adrenocortical tissues. The expression of *ESM1* from **(A)** GSE90713 and **(B)** GSE19750. Relative expression of *ESM1* in ACC, normal tissues **(C)**, and in tumor stages I–IV **(D)** based on GEPIA online server. *p < 0.05, ***p < 0.001.

Furthermore, these results were validated in public database, including GEO and TCGA datasets, for ACC patients. The mRNA expression of *ESM1* was dramatically overexpressed in GSE90713 (*p* = 0.0004, **Figure 2A**) and GSE19750 (*p* = 0.0002, **Figure 2B**). Moreover, correlation analysis by the GEPIA database showed that the mRNA expression of *ESM1* was evidently overexpressed in ACC tumors compared with that in normal ones (*p* < 0.005, **Figure 2C**) but not significantly correlated with the tumor stage for ACC (*p* > 0.05, **Figure 2D**).

### 3.2 Signature Analysis of *ESM1* in Clinicopathological Parameters for ACC Patients

**Figure 3:**
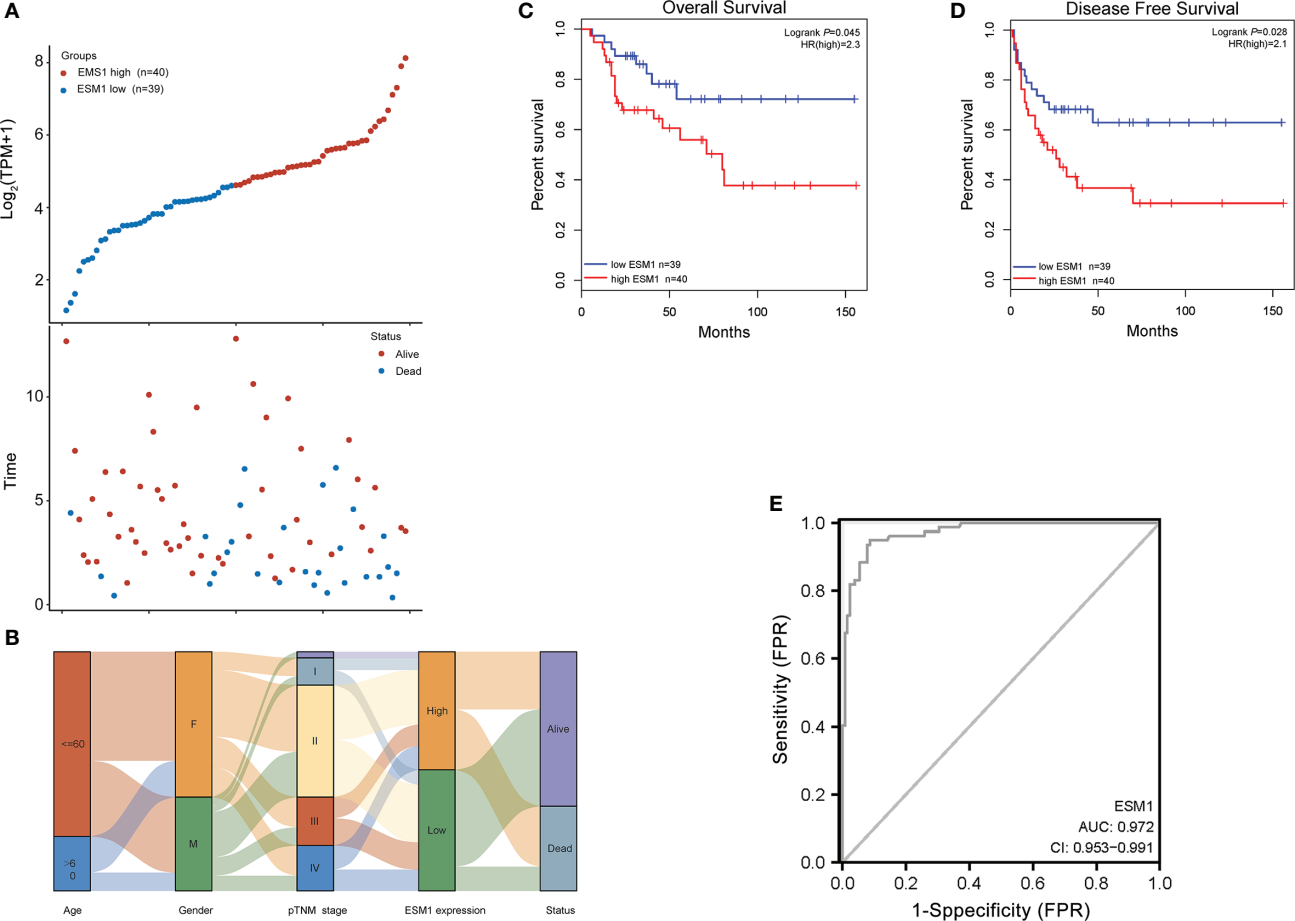
Prognosis analysis of *ESM1* in clinicopathological parameters for ACC patients. **(A)** *ESM1* expression (high or low) and survival status (alive or dead) of ACC patients. The top part denotes the scatter plot of the *ESM1* expression (low to high); the bottom part means the scatter plot distribution of survival time and survival status corresponding to gene expression of each sample; all the sample order is consistent. **(B)** Sanguini diagram for depicting the distribution of *ESM1* expression in age, gender, pTNM stage, and survival status. Each column displays a feature variable, different colors represent different types or stages, and lines represent the distribution of the same sample in different feature variables. The OS **(C)** and PFS **(D)** for patients with high (red) and low (blue) *ESM1* expression in ACC at the threshold of *p*-value <0.05. **(E)** ROC curves of *ESM1* expression in ACC. The area value under the ROC curve is between 0.5 and 1. The closer the AUC is to 1, the better the diagnostic effect. AUC has certain accuracy when it is 0.7~0.9, and higher accuracy when AUC is above 0.9. FPR, false-positive rate; TPR, true-positive rate.

**Figure 4:**
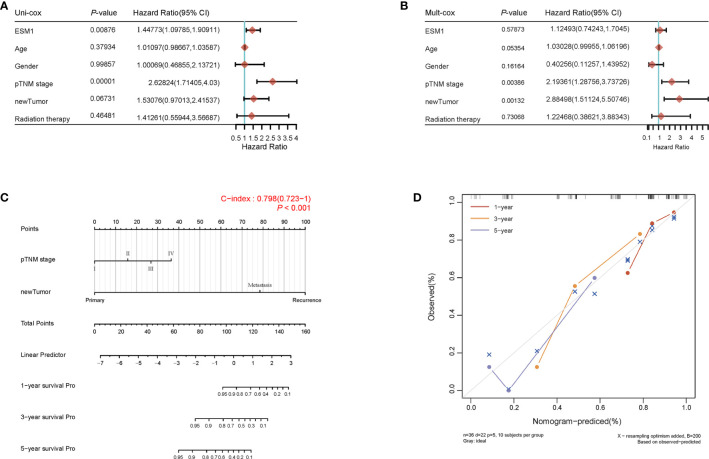
Prognosis analysis of *ESM1* expression in ACC patients. The *p*-value and hazard ratio of *ESM1* and associated parameters of the uni-Cox **(A)** and multi-Cox **(B)** analyses. **(C)** Nomogram for assessing 1-, 2-, and 3-year survival rate in ACC patients related to *ESM1* expression. The nomogram provided a graphical representation of the factors, which can be used to calculate the risk of recurrence for an individual patient by the points associated with each risk factor. **(D)** The calibration curve of the nomogram. The closer the nomogram model is to the calibration curve, the better the prediction result of the model.

For figuring out the correlation between *ESM1* expression and clinicopathological parameters in ACC, all ACC patients were divided into two subgroups, including 39 ESM1^low^ and 40 ESM1^high^ samples according to the median cutoffs (**Figure 3A**). As presented in **Table 1**, the expression of *ESM1* was not correlated with age, gender, laterality, mitotane therapy, and pathological stages (*p* > 0.05). In new tumor events, more ACC cases with ESM1^high^ were prone to lose the chance of surgery or relapse than ESM1^low^ (*p* = 0.022). Moreover, the sanguini diagram described the distribution of *ESM1* expression in age, gender, and pTNM stage (**Figure 3B**). According to the survival rate analysis, upregulation of *ESM1* expression predicts dismal OS (HR = 2.3, log rank *p* = 0.045) and PFS (HR = 2.1, log rank *p* = 0.028) in ACC (**Figures 3C, D**). The ROC curve analysis demonstrated that the *ESM1* had high accuracy in predicting or diagnosing the prognosis of ACC patients (**Figure 3E**, AUC = 0.972, CI = 0.953–0.991). Furthermore, the correlation between *ESM1* and relevant clinical parameters, including age, gender, pTNM stage, etc. on the prognosis of ACC patients were identified *via* the uni-Cox and multi-Cox regression analysis. As a result, the *ESM1* expression and pTNM stage were closely related to the prognosis of ACC patients in uni-Cox analysis (**Figure 4A**, all *p* < 0.05), while the *ESM1* expression could not be served as independent prognostic factors for ACC patients in multi-Cox analysis (**Figure 4B**, all *p* > 0.05). Lastly, 1-, 2-, or 3-year survival rate in one ACC patients related to high *ESM1* expression was assessed by nomogram (**Figures 4C, D**).

**Table 1:** Correlation between *ESM1* expression and clinicopathological characteristics.

Characteristic	ESM1 expression		*p*-Value	Characteristic	ESM1 expression		*p*-Value
	Low	High			Low	High	
Age (mean ± SD)	50.15 ± 15.37	43.33 ± 15.61	0.054	R0	28 (40%)	27 (38.6%)	
Gender [*n* (%)]			0.711	R1	3 (4.3%)	3 (4.3%)	
Female	25 (31.6%)	23 (29.1%)		R2	4 (5.7%)	5 (7.1%)	
Male	14 (17.7%)	17 (21.5%)		Laterality [*n* (%)]			0.436
Pathologic stage [*n* (%)]			0.947	Left	20 (25.3%)	25 (31.6%)	
Stage I	5 (6.5%)	4 (5.2%)		Right	19 (24.1%)	15 (19%)	
Stage II	18 (23.4%)	19 (24.7%)		Mitotane therapy [*n* (%)]			0.195
Stage III	9 (11.7%)	7 (9.1%)		No	16 (21.3%)	10 (13.3%)	
Stage IV	7 (9.1%)	8 (10.4%)		Yes	21 (28%)	28 (37.3%)	
Tumor status [*n* (%)]			0.138	OS event [*n* (%)]			0.012
Tumor free	23 (29.9%)	16 (20.8%)		Alive	31 (39.2%)	20 (25.3%)	
With tumor	15 (19.5%)	23 (29.9%)		Dead	8 (10.1%)	20 (25.3%)	
New event [*n* (%)]			0.022	DSS event [*n* (%)]			0.010
No	25 (32.9%)	14 (18.4%)		Alive	31 (40.3%)	20 (26%)	
Yes	13 (17.1%)	24 (31.6%)		Dead	7 (9.1%)	19 (24.7%)	
Residual tumor [*n* (%)]			1.00				

### 3.3 GSEA Analyses Reveal Masked Molecular Mechanisms of *ESM1* in Tumorigenesis and Progression

**Figure 5:**
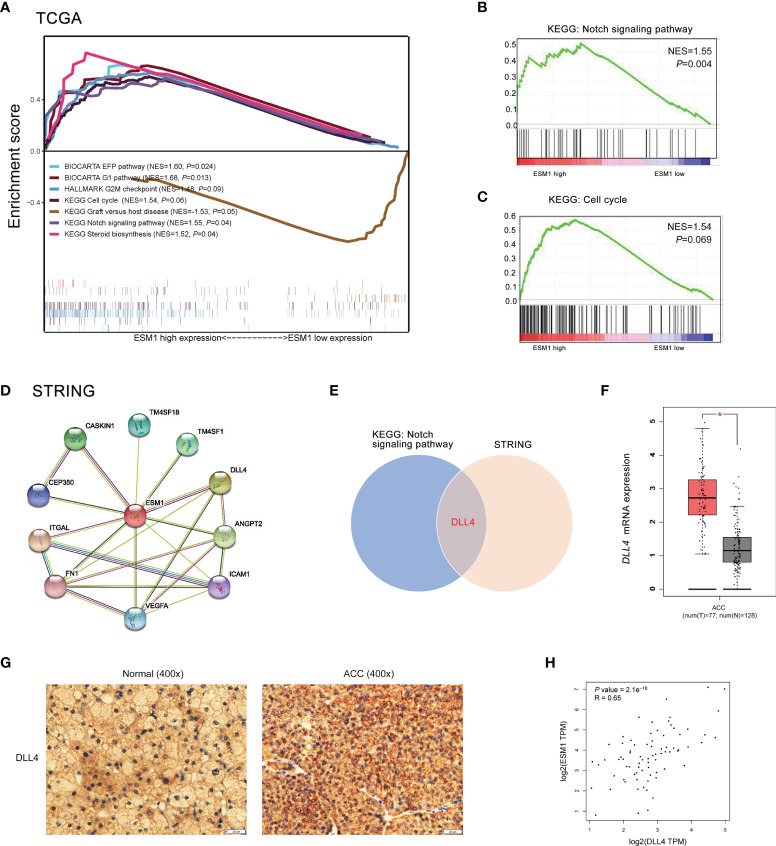
The GSEA analysis of potential signaling pathway in ACC patients with ESM1^high^ *versus* ESM1^low^. **(A)** The GSEA analysis of KEGG, Biocarta, Hallmark enrichment pathway in ACC patients with the NES and *p*-values of corresponding pathways. The Notch signaling pathway **(B)** and cell cycle **(C)** was significantly enriched in GSEA analyses. **(D)** The protein-protein interaction network of ESM1 and related proteins based on STRING platform. **(E)** Venn diagram of Notch signaling pathway and STRING analyses. DLL4 expression based on GEPIA platform **(F)** and IHC of human samples **(G)**. **(H)** The correlation between the expression of ESM1 and DLL4 in ACC. *p < 0.05.

**Figure 6:**
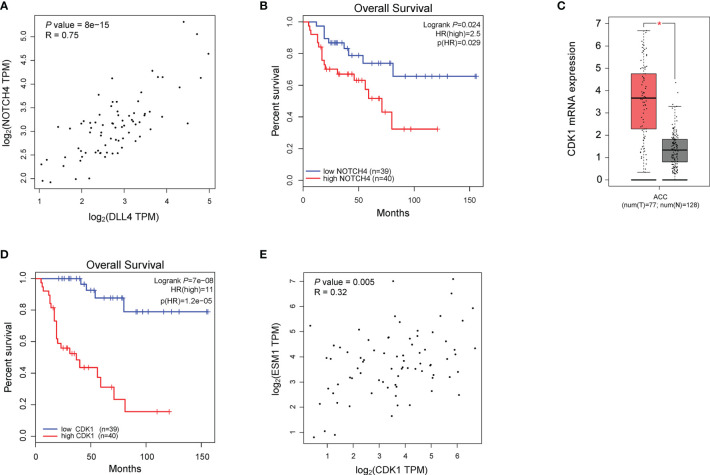
The analysis of potential signaling pathway in ACC patients with ESM1^high^ *versus* ESM1^low^. **(A)** The correlation between the expression of *DLL4* and Notch4 in ACC. **(B)** The OS of ACC patients with Notch4 high or low expression. The expression **(C)** and OS **(D)** of *CDK1* in ACC patients. **(E)** The correlation between the expression of ESM1 and CDK1 in ACC. *p < 0.05.

To reveal the underlying roles of *ESM1* in cancer-related signaling pathways, GSEA was performed to interpret the gene expression profiles of ACC specimens in ESM1^low^ and ESM1^high^ group based on TCGA dataset. According to GSEA analysis of KEGG pathway, ACC patients with ESM1^high^ were mainly enriched in cell cycle and Notch signaling pathway (**Figures 5A–C**). The analysis of Biocarta and Hallmark pathways demonstrated that ACC patients with ESM1^high^ were mostly involved in G1 pathway and G2/M checkpoint, respectively (**Figure 5A**). Venn Diagram of Notch signaling pathway and PPI network of STRING analyses (**Figure 5D**) displayed that DLL4 was a key protein, which closely interacted with ESM1 and was mainly involved in Notch signaling pathway (**Figure 5E**). Furthermore, the expression of *DLL4* was notably upregulated based in ACC on GEPIA platform (**Figure 5F**, *p* < 0.05) and human samples (**Figure 5G**) and positively correlated with the expression of ESM1 (**Figure 5H**; *R* = 0.65, *p* < 0.001). Moreover, the expression of *DLL4* was markedly related to Notch4 in ACC samples (**Figure 6A**; *R* = 0.75, *p* < 0.001), which suggested a dismal OS in ACC (**Figure 6B**, *p* = 0.024). As one of the vital regulators in cell cycle, *CDK1* was dramatically overexpressed (**Figure 6C**, *p* < 0.05), suggested a poor OS (**Figure 6D**, *p* < 0.001), and was positively correlated with the expression of *ESM1* (**Figure 6E**; *R* = 0.32, *p* = 0.005).

### 3.4 *ESM1* Interference Influences the Cell Proliferation, Migration, Cell Cycle, and Apoptosis of ACC

**Figure 7:**
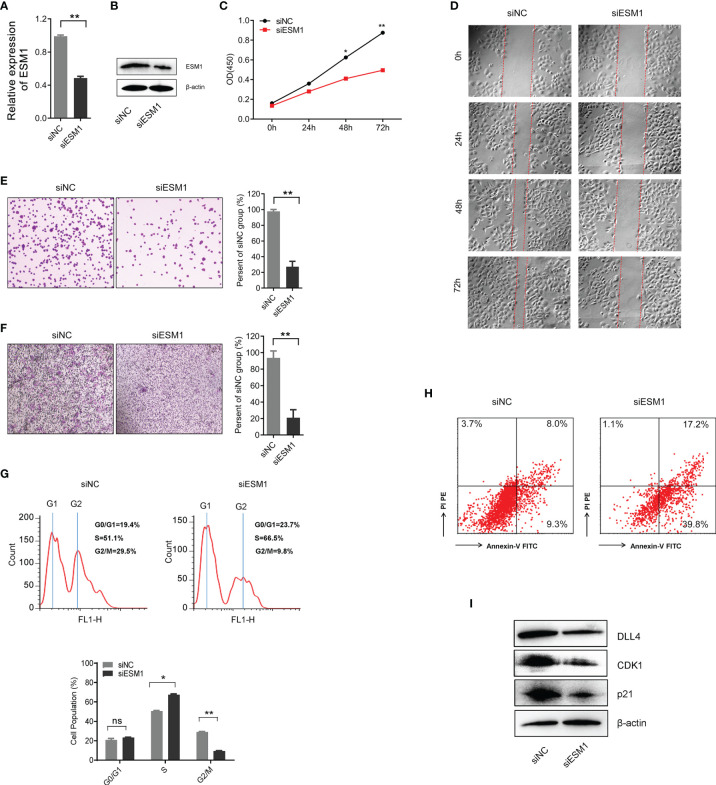
*ESM1* might be involved in cell proliferation, cell migration, cell cycle, and cell apoptosis in human SW13 cells. Detection method by **(A)** the qRT-PCR and **(B)** Western blotting for ESM1 expression in human SW13 cells after transfected with siESM1 or siNC for 72 h. **(C)** Cell proliferation assay for human SW13 cells after cells were transfected with siESM1 or siNC for 72 h. **(D)** Wound-healing experiment for cell mobility investigation. The red line denotes the migration ability of SW13 cells transfected with siESM1 or siNC for 48 hours. **(E)** Cell adhesion assay of SW13 cells transfected with siESM1or siNC for 48 h. **(F)** Cell invasion assay of SW13 cells transfected with siESM1 or siNC for 48 h. **(G)** Cell cycle analysis and **(H)** apoptosis of SW13 cells transfected with siESM1 or siNC for 48 h. **(I)** Western blotting for DLL4, CDK1, and p21 in human SW13 cells transfected with siESM1 or siNC for 48 h. ns denotes no significant difference, *p < 0.05, **p < 0.01.

In order to explore the role of *ESM1* in ACC, *ESM1* siRNAs (siESM1) were constructed *via* ACC cell line, human SW13 cells, as *in vitro* experiments. The expression of *ESM1* was overtly inhibited in siESM1, compared with siNC (**Figures 7A, B**). In cell proliferation assay, *ESM1* interference evidently inhibited cell proliferation (**Figure 7C**) and cell migration (**Figure 7D**) in human SW13 cells after cells were transfected for 72 h. Inhibition of *ESM1* distinctly reduced adhesion between tumor cells and matrix (**Figure 7E**) and cell invasion (**Figure 7F**) in human SW13 cells. In addition, flow cytometry assay displayed that *ESM1* interference induced cell cycle arrest at G2/M-phase transition in human SW13 cells (29.5% *vs*. 9.8%, **Figure 7G**). Downregulation of *ESM1* overtly intensified cell apoptosis (8.0% *vs*. 17.2%, **Figure 7H**). As mentioned above, the GSEA enrichment analysis uncovered that Notch signaling pathway might serve as a masked molecular mechanism of *ESM1* involved in the occurrence and development of human ACC (**Figures 5A, B**). The expression of *DLL4* was then closely related to *ESM1* in ACC (**Figure 5H**). Moreover, CDK1 and p21 were downstream-targeted proteins as Notch signaling pathway was activated. Thus, the expression of DLL4, CDK1, and p21 were detected by Western blotting in human SW13 cells. As the interference of *ESM1* by siRNA, the expression of DLL4, CDK1, and p21 were significantly downregulated compared with the siNC in SW13 cells (**Figure 7I**). Therefore, inhibition of *ESM1* decreased the expression of DLL4, CDK1, and p21 and altered downstream protein levels, including apoptosis and cell cycle-related proteins.

### 3.5 The Potential Therapeutic Strategies or Available Chemical Drugs for the Treatment of ACC

**Figure 8:**
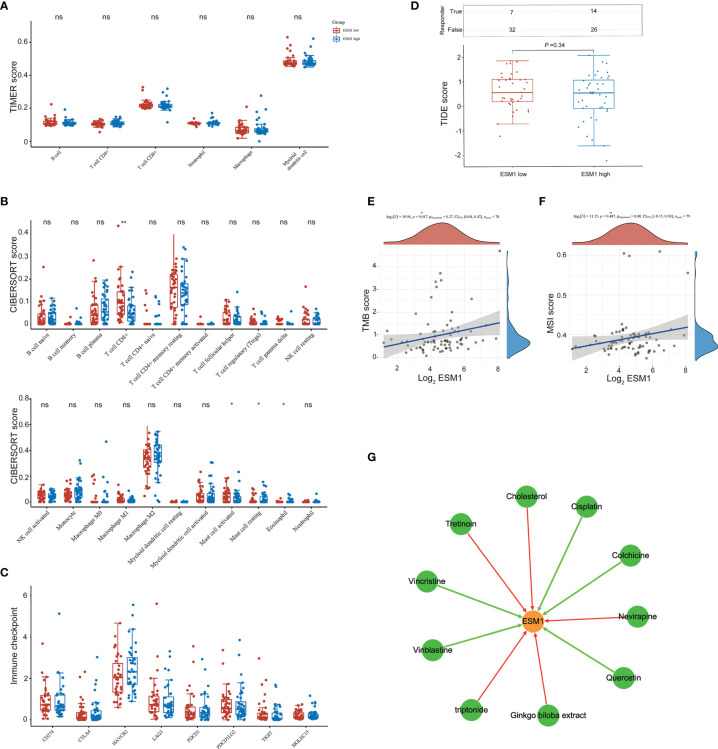
The potential therapeutic strategies or available chemical drugs for the treatment of ACC. **(A)** Immune infiltration analysis, including TIMER score **(A)**, CIBERSORT score **(B)**, immune checkpoint-related genes **(C)**, TIDE score **(D)**, TMB score **(E)**, and MSI score **(F)**, of two subgroups including ESM1^high^ (*n* = 40) and ESM1^low^ (*n* = 39) samples in ACC patients. TIDE, tumor immune dysfunction and exclusion. The horizontal axis represents the gene expression distribution, and the vertical axis denotes the expression distribution of TMB/MSI score. The density curve on the right side represents the distribution trend of TMB/MSI score. The density curve of the upper part represents the distribution trend of genes. The value represents the correlation *p*-value, correlation coefficient, and calculation method. **(G)** The gene-drug interaction network of ESM1 and related chemotherapeutic drugs. Red arrows: chemotherapeutic drugs upregulate ESM1 expression; green arrows: chemotherapeutic drugs downregulate ESM1 expression. ns denotes no significant difference, *p < 0.05, **p < 0.01.

We then further studied the available chemical drugs and treatment strategies (such as immunotherapy) that may be favorable for the treatment of ACC. Firstly, the characterizations of immune cell infiltration in ACC with ESM1^high^ and ESM1^low^ were performed to investigate tumor immunotherapy. All the 79 ACC tissues from TCGA database were divided to two groups, including 40 ESM1^high^ and 39 ESM1^low^ ACC tissues by median cutoffs, and normalized by ‘Limma’ packages. TIMER online platform and CIBERSORT software were applied to analyze immune cell infiltration, which may relate to the occurrence and development of ACC with low and high expression of *ESM1*. The statistical threshold was set as *p* < 0.05. However, no significant difference was revealed between in ACC with ESM1^high^ and ESM1^low^ according to the analysis of TIMER (**Figure 8A**) and CIBERSORT (**Figure 8B**). Immune checkpoint-related genes, including *CD274*(*PD-L1)*, *CTLA4, HAVCR2*, *LAG3*, *PDCD-1*(*PD-1)*, *PDCD1LG2*(*PD-L2)*, *TIGIT*, and *SIGLEC15(CD33L3)* were investigated to show that no significant difference was found between ACC with ESM1^high^ and ESM1^low^ (**Figure 8C**). The analysis of immune checkpoint blocking (ICB) response suggested that the TIDE score between the ESM1^high^ and ESM1^low^ group had no significant difference (**Figure 8D**). Moreover, the correlation between *ESM1* expression and MSI or TMB were analyzed to unraveled that the expression of *ESM1* was significantly associated with TMB (**Figure 8E**, *p* = 0.017) but not MSI (**Figure 8F**, *p* > 0.05) in ACC. It hinted that overexpression of *ESM1* augments the accumulation of abnormal gene mutation, namely TMB, during cell cycle of DNA replication in ACC. Then, the gene-drug interaction network indicated that a variety of drugs could affect the expression levels of *ESM1* in mRNA or protein level (**Figure 8G**). For example, cisplatin, colchicine, quercetin, vinblastine, and vincristine could inhibit *ESM1* expression while tretinoin, cholesterol, etc. could induce *ESM1* expression. Consequently, all these *ESM1* inhibitors may serve as potential targets for the therapy of ACC.

## 4 Discussion

Multiple literatures have shown that the aberrant expression of *ESM1* frequently occurred in multiple malignant neoplasms. Julien et al. reported that *ESM1* served as a reliable biomarker of macrotrabecular-massive hepatocellular carcinoma (19). Cai et al. suggested that *ESM1* was downregulated *via* exosome microRNA-9-3p to block the development of bladder cancer (20). Xu et al. asserted that *ESM1* was overexpressed in HNSCC and correlated with angiopoietin-2 (ANGPT2) (21). Wang revealed that *ESM1* might act as a novel prognostic signature of immune-related genes for patients with colorectal cancer (22). Gu X figured out that ESM1/HIF−1α pathway appeared to be pivotal mediator of chronic intermittent hypoxia−induced lung cancer progression (23). Nevertheless, the expression alteration and exact roles of *ESM1* in ACC patients remain undefined, and the molecular mechanisms and functions of *ESM1* are still unclear. This study intended to systematically explore the expression patterns, prognosis, and latent functions of *ESM1* in ACC.

In the current study, the IHC staining suggested that ESM1 was overtly overexpressed in ACC patients. Specifically, ESM1 was strongly expressed in the cytoplasm and membrane of ACC. In adrenocortical adenomas, ESM1 has a strong cytoplasmic expression and weak membrane expression. Whereas, it was weakly or moderately expressed in the cytoplasm of normal adrenal cortex cells. It indicated that ESM1 might exert its biological function through secreting massive ESM1 protein from the cytoplasm to the cell membrane in the process of carcinogenesis for ACC patients.

The further insight into the expression of *ESM1* was gained *via* assaying ACC datasets from GEO and TCGA. It confirmed that *ESM1* was upregulated in ACC, compared with normal ones. According to the uni-Cox analysis, the *ESM1* expression and pTNM stage were correlated with the prognosis of ACC patients. Thus, it suggested that *ESM1* could be viewed as a biomarker to make a distinction between ACC and adrenocortical adenoma or normal adrenal cortex tissues. Then, prognosis of *ESM1* were evaluated to announce that overexpression of *ESM1* were overtly related to the deterioration of OS and PFS in ACC patients. It asserted that up-regulation of *ESM1* was a hazard factor for the prognosis of ACC. The ROC curve analysis revealed that the *ESM1* had high accuracy in predicting or diagnosing the prognosis of ACC patients (AUC = 0.972). A recent study identified that overexpressed *ESM1* exerted as a novel oncogene for esophageal cancer (25). Concurrently, our results showed that the expression of *ESM1* was upregulated in ACC and might play a crucial role in the tumorigenesis and progress of ACC.

According to the GSEA analysis of KEGG pathway, ESM1^high^ was indicated to be mainly involved in Notch signaling pathway and cell cycle. Based on PPI network, ESM1 interacts closely with DLL4 and ANGPT2.While DLL4 is one of the important ligands of Notch signaling pathway (43). Additionally, the expression of *ESM1* was highly positively correlated with the *DLL4* expression. Thus, ESM1 might interact closely with DLL4 and involved in Notch signaling pathway during the occurrence and development of ACC. Moreover, Li et al. suggested cyclin D1, p21, etc. as target genes of Notch signaling pathway downstream (43). As one of the vital regulators in cell cycle, *CDK1* was dramatically overexpressed, suggested a poor OS, and positively correlated with the expression of *ESM1*. Thus, we hypothesized that EMS1/DLL4-Notch signaling axis might regulate cell cycle by interacting with CDK1 and p21 in human ACC.

Based on the above results, *in vitro* experiment of siRNA system was designed to probe potential biological functions and molecular mechanisms by conducting of *ESM1* in human ACC cell line, SW13 cells. The results asserted that inhibition of *ESM1* expression impeded the cell proliferation, adhesion between tumor cells and matrix, cell migration, cell invasion, and G2/M-phase transition, while remarkably expedited cell apoptosis of SW13 cells. These results suggested that *ESM1* might induce G2/M-phase transition by interacting with *CDK1*, which played a vital role in promoting cell proliferation. Additionally, the overexpression of *ESM1* induced cell migration, cell invasion, and inhibiting cell apoptosis. By Western botting, interference of *ESM1* by siRNA, the expression level of DLL4, CDK1, and p21 were decreased compared with the siNC in SW13 cells. Therefore, the *ESM1* augmented the expression level of DLL4, activated Notch signaling pathway, and altered downstream protein levels, including CDK1 and p21 that were related to cell cycle regulation.

Previous literature reports enhanced our results. Kojima et al. demonstrated that CDK1 inhibitor enhanced p53-mediated mitochondrial apoptosis by Bax activation and G2/M-phase cell cycle arrest in acute myeloid leukemia (44). Danupon et al. reported that CCNB1/CDK1 complex were relocated to mitochondria during G2/M-phase arrest in HCT116 cells (45). Abnormal mitosis induced by CCNB1/CDK1 complex is an enormous element of cancer development or progression (46). Combined with previous related research, we draw the conclusion that overexpression of *ESM1* upregulated CDK1 or p21-mediated G2/M-phase transition, cell proliferation, cell migration, and invasion *via* DLL4-Notch signaling pathway.

Even though multiple biomarkers and potential molecular mechanisms of ACC have been reported, effective drugs for the therapy of ACC are still scanty (47). Therefore, the potential target genes or immunotherapy strategies associated with high expression of *ESM1* were further investigated. Firstly, as one of the hallmarks of cancer, immune microenvironment has become a hot point in multiple cancer research (48). In the current study, immune cell infiltration of ACC with *ESM1* high and low expression was analyzed by TIMER and CIBERSORT platforms to reveal that the cell types were not evidently different in ACC samples of ESM1^high^, compared with ESM1^low^. Moreover, patients with high *ESM1* expression characterized higher TMB but not MSI. In addition, the mRNA expression of immune checkpoint-related genes, including *CD274*(*PD-L1)*, *CTLA4*, *HAVCR2*, *LAG3*, *PDCD-1*(*PD-1)*, *PDCD1LG2*(*PD-L2)*, *TIGIT*, and *SIGLEC15(CD33L3)*, were not different in ACC samples of the ESM1^high^ group and ESM1^low^ group. These analyses of immune checkpoint-related genes suggested that no notable difference was found in ACC patients between EMS1^high^ and EMS1^low^ groups. The overexpression of *ESM1* might trigger accumulation of tumor mutation burden (TMB) during cell cycle of DNA replication in ACC. Moreover, gene-drug interaction network was constructed by CTD to find that multiple *ESM1* inhibitors, including cisplatin, colchicine, quercetin, vinblastine, and vincristine, might serve as potential targets for the therapy of ACC patients with *ESM1* high expression. Nevertheless, further molecular mechanisms and pharmaceutical or clinical practice are still required to verify these hypotheses.

**Figure 9:**
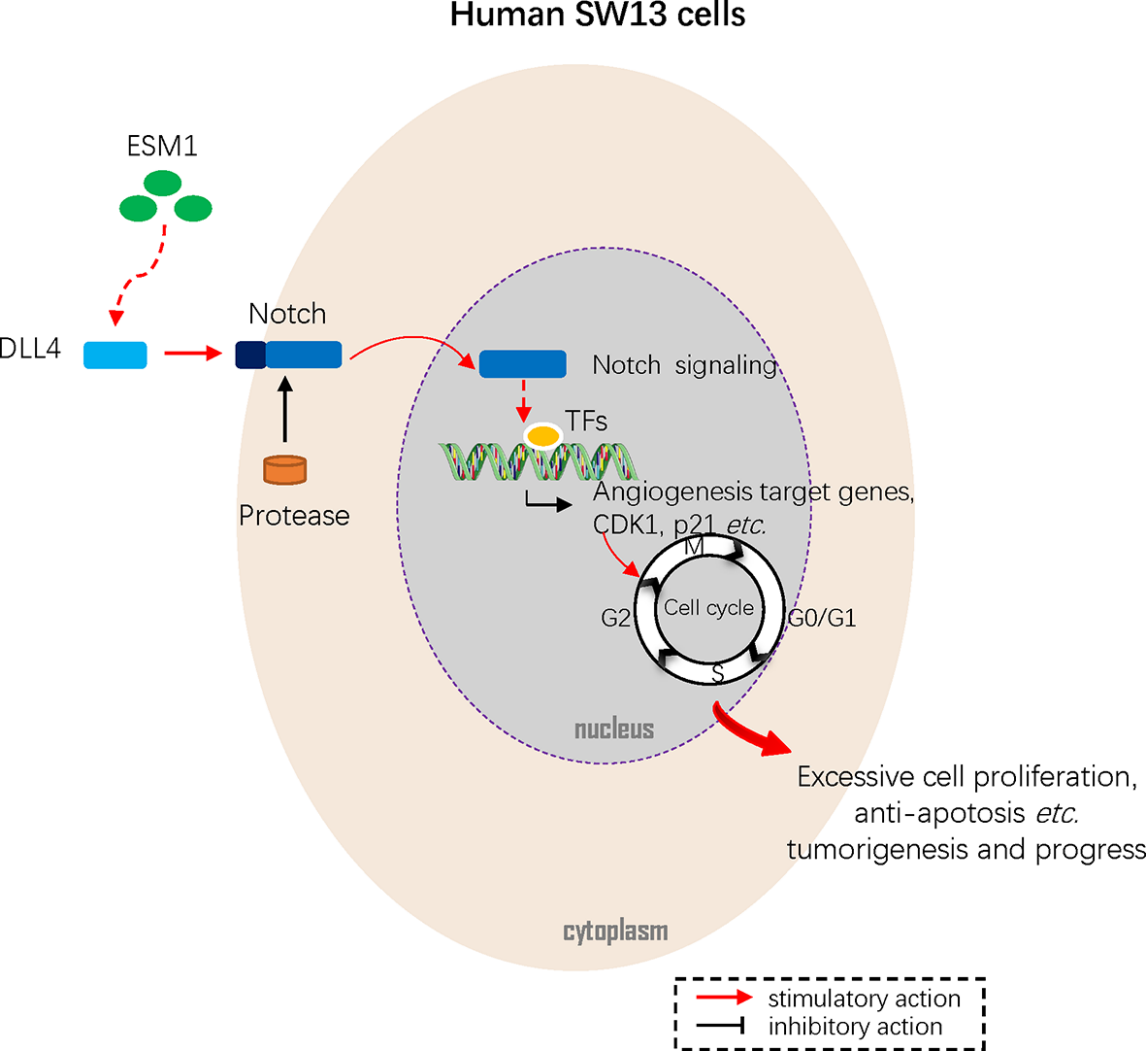
Mechanism diagram. Overexpressed ESM1 upregulated CDK1 and p21 mediated G2/M-phase transition, cell proliferation, cell migration, cell invasion, and accumulation of tumor mutation burden (TMB) *via* DLL4-Notch signaling pathway.

In summary, this study figured out that *ESM1* is significantly overexpressed in ACC in terms of mRNA and protein. The overexpressed *ESM1* predicted an unfavorable prognosis of ACC and might contribute to the initiation and progress of ACC. The results suggested that *ESM1* might serve as a potential prognostic biomarker or therapeutic target for ACC patients. According to *in vitro* experiments of human ACC cell line, SW13 cells, overexpression of *ESM1* upregulated *CDK1* and p21-mediated G2/M-phase transition, cell proliferation, cell migration, cell invasion, and accumulation of tumor mutation burden (TMB) *via* DLL4-Notch signaling pathway (**Figure 9**). Collectively, the study threw light on the accumulating evidence about ESM1 and relevant signaling pathways, which might furnish clues for the development of *ESM1*-mediated therapeutic drugs or strategies for ACC.

## Data Availability Statement

All publicly available datasets analyzed in this study can be acquired from GEO (https://www.ncbi.nlm.nih.gov/geo/) and TCGA (https://portal.gdc.cancer.gov/).

## Ethics Statement

The studies involving human participants were reviewed and approved by the Ethics Committee of Taihe Hospital. The patients/participants provided their written informed consent to participate in this study.

## Author Contributions

Y-GH and YW conceived, designed, performed statistical analysis, and wrote the paper. X-MS and X-BT supervised the research. X-MS revised the manuscript. R-JZ participated in the study design. KT provisioned useful suggestions in methodology and figure preparation. All authors contributed to the article and approved the submitted version.

## Funding

National Natural Science Foundation of China, Grant/Award Number: 81600436.

## Conflict of Interest

The authors declare that the research was conducted in the absence of any commercial or financial relationships that could be construed as a potential conflict of interest.

## Publisher’s Note

All claims expressed in this article are solely those of the authors and do not necessarily represent those of their affiliated organizations, or those of the publisher, the editors and the reviewers. Any product that may be evaluated in this article, or claim that may be made by its manufacturer, is not guaranteed or endorsed by the publisher.
