# Supplementary material for: EMS1/DLL4-Notch Signaling Axis Augments Cell Cycle-Mediated Tumorigenesis and Progress in Human Adrenocortical Carcinoma
Source: Front Oncol. 2021 Nov 10;11:771579. doi: 10.3389/fonc.2021.771579 (PMC8631517; doi:10.3389/fonc.2021.771579)
Supplement: Supplementary file 1 [file Table_1.docx]

**Table S1. Details of all patients involved in this study.**

| **Category** | **Gender** | **Age** | **Tumor location** | **Tumor metastasis** | **Year of diagnosis** |
| --- | --- | --- | --- | --- | --- |
| AA-1 and Normal-1 | Female | 51 | Left adrenal gland | No | 2020 |
| AA-2 and Normal-2 | Female | 52 | Right adrenal gland | No | 2020 |
| AA-3 and Normal-3 | Female | 53 | Right adrenal gland | No | 2020 |
| AA-4 and Normal-4 | Male | 41 | Right adrenal gland | No | 2020 |
| AA-5 and Normal-5 | Female | 57 | Right adrenal gland | No | 2020 |
| AA-6 and Normal-6 | Female | 51 | Right adrenal gland | No | 2020 |
| AA-7 and Normal-7 | Female | 52 | Left adrenal gland | No | 2020 |
| AA-8 and Normal-8 | Male | 38 | Right adrenal gland | No | 2020 |
| AA-9 and Normal-9 | Male | 54 | Right adrenal gland | No | 2020 |
| AA-10 and Normal-10 | Female | 45 | Right adrenal gland | No | 2020 |
| AA-11 and Normal-11 | Male | 55 | Right adrenal gland | No | 2020 |
| AA-12 and Normal-12 | Female | 62 | Right adrenal gland | No | 2020 |
| ACC-1 | Male | 25 | Right adrenal gland | No | 2013 |
| ACC-2 | Female | 44 | Left adrenal gland | No | 2013 |
| ACC-3 | Male | 59 | Right adrenal gland | No | 2013 |
| ACC-4 | Male | 53 | Left adrenal gland | No | 2019 |
| ACC-5 | Female | 34 | Left adrenal gland | With lung metastasis | 2013 |
| ACC-6 | Male | 53 | Left adrenal gland | No | 2018 |

Adrenocortical adenoma: AA; Adrenocortical Carcinoma: ACC.
