# Supplementary material for: EMS1/DLL4-Notch Signaling Axis Augments Cell Cycle-Mediated Tumorigenesis and Progress in Human Adrenocortical Carcinoma
Source: Front Oncol. 2021 Nov 10;11:771579. doi: 10.3389/fonc.2021.771579 (PMC8631517; doi:10.3389/fonc.2021.771579)
Supplement: Supplementary file 2 [file Table_2.docx]

**Table S2. Details of all antibodies involved in this study.**

| **Antibodies for western blotting** | **Source** | **Catalogs NO.** | **Molecular weight** |
| --- | --- | --- | --- |
| Anti-ESM1 antibody | Cloud-Clone Corp. | Cat: PAC463Hu01 | 19kDa |
| Anti-CDK1 antibody | ZEN BIO | Cat:383884 | 34 kDa |
| Anti-DLL4 antibody | Proteintech | Cat:21584-1-AP | 75~80 kDa |
| Anti- p21 antibody | Proteintech | Cat:10355-1-AP | 21 kDa |
| β-actin antibody | Santa Cruz | Cat:sc-47778 | 42 kDa |
