# Supplementary material for: EMS1/DLL4-Notch Signaling Axis Augments Cell Cycle-Mediated Tumorigenesis and Progress in Human Adrenocortical Carcinoma
Source: Front Oncol. 2021 Nov 10;11:771579. doi: 10.3389/fonc.2021.771579 (PMC8631517; doi:10.3389/fonc.2021.771579)
Supplement: Supplementary file 3 [file Table_3.docx]

**Table S3 Details of ACC studies and associated microarray datasets from GEO database.**

| **GEO Series** | **Contributor(s)** | **Sample** | | **Platform** | **Submission time** | **Country** |
| --- | --- | --- | --- | --- | --- | --- |
|  |  | **Tumor** | **Normal** |  |  |  |
| GSE90713 | Farber JM et al, 2016 | 58 | 5 | GPL15207 Affymetrix Human Gene Expression Array | 2016 | USA |
| GSE19750 | Bussey KJ et al, 2010 | 44 | 4 | GPL570 Affymetrix Human Genome U133 Plus 2.0 Array | 2010 | USA |
