# Supplementary material for: EMS1/DLL4-Notch Signaling Axis Augments Cell Cycle-Mediated Tumorigenesis and Progress in Human Adrenocortical Carcinoma
Source: Front Oncol. 2021 Nov 10;11:771579. doi: 10.3389/fonc.2021.771579 (PMC8631517; doi:10.3389/fonc.2021.771579)
Supplement: Supplementary file 4 [file Table_4.docx]

**Table S4.** The sequences information of qRT-PCR primers and siRNAs.

| **Gene name** | **Primers (5**'-**3**') | |
| --- | --- | --- |
| ***ESM1*** | Forward: CTTGCTACCGCACAGTCTCA | Reverse: GCCATGTCATGCTCTTTGCAG |
| ***GAPDH*** | Forward: CGGAGTCAACGGATTTGGTCGTAT | Reverse: AGCCTTCTCCATGGTGGTGAAGAC |
| **siESM1** | Forward: ACUUGUAUGUGUUUGUUAAAU | Reverse: UUAACAAACACAUACAAGUGU |
| **siNC** | Forward: UUCUCCGAACGUGUCACGUTT | Reverse: ACGUGACACGUUCGGAGAATT |

siESM1: *ESM1* siRNA, siNC: negative control siRNA.
